# Path Dependence and Universal Health Coverage: The Case of Egypt

**DOI:** 10.3389/fpubh.2017.00325

**Published:** 2017-12-08

**Authors:** Ayman Fouda, Francesco Paolucci

**Affiliations:** ^1^Department of Economics, School of Economics, Management and Statistics, University of Bologna, Bologna, Italy; ^2^School of Law, Rotterdam Institute of Law and Economics, Erasmus University Rotterdam, Rotterdam, Netherlands; ^3^Sir Walter Murdoch School of Public Policy and International Affairs, Murdoch University, Perth, WA, Australia

**Keywords:** path dependence, institutional settings, health systems, egypt, universal health coverage

## Abstract

Universal health coverage (UHC) is the big objective in health policy which several countries are seeking to achieve. Egypt is no different and its endeavors to attain UHC have been going on since the 1960s. This article discusses the status of UHC in Egypt using theories of political science and economics by analyzing the historical transformations in the Egyptian health system and its institutional settings. This article then specifically examines the path dependence theory against the sociopolitical background of Egypt and assesses any pattern between the theory and the current UHC status in Egypt. The important finding of this analysis is that the health policies and reforms in Egypt have been significantly influenced and limited by its historical institutional structure and development. Both the health policies and the institutional settings adopted a dependent path that limited Egypt’s endeavors to achieve the universal coverage. This dependent path also yielded many of the present-day challenges as in the weaknesses of the healthcare financing system and the inability to extend health coverage to the poor and the informal sector. These challenges subsequently had a negative impact on the accessibility of the healthcare services.

## Introduction

Universal health coverage (UHC), the big term in health policy, is the ultimate aim for several countries and intergovernmental organizations such as the World Health Organization. The endeavor to achieve UHC is known to be sensitive to the historical and political alterations, which can largely shape and affect this endeavor in many countries. These political alterations can affect the endeavor of UHC by limiting it to a stiff and rigid trajectory that might end up in shortage of the main objective. In attempts to understand and depict these trajectories, path dependence theory emerges as a potential explanatory tool on how historical events can affect the present and the future.

Universal health coverage, political transformations, and path dependence are the three big pillars which this article studies and connects in the context of the Egyptian health system. This article is structured in two main sections: “Section (1): Path Dependence Theory” discusses the characteristics, framework, and application of path dependence theory and is followed by “Section (2): The Sociopolitical Alterations and the Health Sector in Egypt,” which describes an analysis of the sociopolitical transformation and its reflection on the health sector and the status of UHC in Egypt. This article ends with a discussion on the findings of both sections under the Discussion and Conclusion sections.

### Section (1): Path Dependence Theory

What makes some organizations or policies persist over time? If there are better alternatives, why can we not select them? In the past few years, several studies have tried to identify and investigate the history of the organizational structure, evolutions in technology, and policy reforms by applying process theories. More specifically, studying the locked-in organizations and policies has gained attention to challenge this locked-in status and disrupt the policy or organizational *status quo*—if this *status quo* did not and will not lead to achieving the policy or organizational goal. In many cases, the *status quo* or the persistence is treated as a starting point or an outcome, but the underlying process that produced this *status quo* or persistence remains unchallenged and underexplored (1).

To challenge the persistence, studying the underlying processes emerges as a main step to provide policymakers and stakeholders with insights regarding the *status quo*. Studying the contextual circumstances of seemingly suboptimal limiting decisions (regarding the formation of organizations or policies) is crucial since these decisions may have shaped the present and may also shape the future. According to Antonelli (2), path dependence theory is adopted to understand how past events or decisions can influence present and future decisions. Path dependence theory is used to describe and explain the dynamics of such processes by incorporating the temporal dimension into the process theory (3).

Path dependence theory has been used in many domains, such as technological development, economics, political sciences, national corporate governance systems, urbanism, tourism, industrial clusters, management operations, scientific knowledge, export behavior, and innovation systems, and recently has been introduced to the health policy field (4). Perhaps the archetypical example of path dependence theory is the persistence of the QWERTY keyboard in new technologies despite the emergence of newly technically superior alternatives (5).

#### Definitions and Characteristics

What is path dependence? The orthodox simple explanation of the path-dependent processes is that “the social world often follows a particular trajectory: an open period during which there are a number of plausible alternatives, a critical juncture where contingent events result in one of these alternatives being selected, and then feedback that constrains actors to keep to that particular path” (6). Another definition that emphasizes the surroundings of the action or decision is that path dependence “is a specific form of complex dynamics: It provides an analytical framework to explain and assess the ever-changing outcomes of the combination of and interaction amongst factors of continuity/discontinuity, growth and development, hysteresis and creativity, routines and ‘free will’, which all characterize economic action in a dynamic perspective that is also able to appreciate the role of historic time” (2, 4). Another important definition for path dependence is that it refers to “a property of contingent, non-reversible dynamic processes, including a wide array of processes that can be properly be described as evolutionary” (7).

According to Mahoney (8), path dependence is a set of consecutive events, which start unfolding from a key choice milestone or critical juncture. It is important to comprehend that critical junctures take place only if there are many alternatives or at least two alternatives. Once one alternative is selected, it imposes progressive limitations, which make it difficult to deviate away from the selected alternative or to return to the 0 point.

Accordingly, three main characteristics can characterize path dependence theory:

1. The critical juncture point must encompass several alternatives or at least two based on the antecedent conditions.
2. The consecutive events are connected and do not occur separately.
3. The outcomes are strongly influenced by the antecedent conditions although these conditions might be non-existent afterward.

#### Application in Healthcare

Path dependence theory has been introduced to the field of health policy to elaborate the current state of some health policy reforms and to shed light on the institutional settings in healthcare. Several studies used different methods of modeling path dependence that have been used in health policy. For example, a comparative analysis between defined health policies in several countries has been examined using path dependence model (9). Another study on the United States’ health and environmental agencies evaluated the choice of organizational form as a problem of institutional settings and its relation to the historical and social conditions from the perspective of the path dependency theory (10). A third example can be found in a study about how the latest healthcare reforms in the United States might shape the future (11).

It is important to note that researchers apply path dependence theory in health policy in two perspectives: as a retrospective analysis of the present situation or as a prospective prediction of the future trajectory. The classic path dependence model (retrospective) assesses and looks back at the past events to find the critical juncture that influenced the alternatives afterward; in other words, it is all about past events and the limitations of the past choices. In contrast, Jacobs and Skocpol (12) presented a different approach, which represents the prospective application of path dependence theory where it was argued that the Affordable Care Act (ACA), which was enacted in 2010 in the United States will serve as a critical juncture for the American health system and its effects or repercussions will be seen in the future. Haeder (11) also adopted this prospective view to analyze the ACA and concluded that “it will create certain lock-in effects that will be difficult to alter in the future because the ACA altered the trajectory of health policy by making access to care as a social right, by widening the arena for public decision making, and by socializing the conflict.” The notion of predicting future consequences based on a present-time critical juncture is considered unconventional compared with the classic model of analyzing past critical junctures and their historical consequences.

#### Debating Path Dependence

Scholars and researchers have been involved in many debates regarding path dependence theory. In particular, the ongoing debates focused on the view and model of application as highlighted below.

##### The Deterministic View vs. the Stochastic View

The debate between the deterministic and stochastic views about path dependence theory revolves around the nature of the process itself. The deterministic view, which is frequently used by scholars, suggests that after selecting one alternative at the phase of critical juncture, it becomes progressively harder to deviate from the trajectory. While the stochastic view assumes that despite the fact that path dependence intends to connect the past with the present and future, antecedent conditions do not solely determine the outcome. A sequence of unpredictable or random events can have greater influence than the antecedent conditions and can lead to new trajectories rather than the contingent expected one (13).

##### The Canonical Model vs. the Developmental Model

This debate between the canonical and the developmental models is mainly confined to the nature of the outcomes (whether it is continuous and persistent or evolutionary). According to the canonical path dependence model, certain outcomes become “locked-in” or entrapped by self-reinforcing mechanisms and these outcomes are contingent on some antecedent conditions (8). The process of entrapment means that the chances of deviating from the path are very narrow or almost non-existent, which seems to suggest that the outcomes are then necessary ones. In other words, the canonical path dependence model apparently represents a model of “contingent-necessity” and emphasizes the “continuity” path rather than the “evolutionary” path (14). In contrast, an opposite model, which dubbed the developmental path dependence model, was debated by Martin (14), who argued that path dependence does not have to lead to “locked in” or contingent outcomes necessarily. Martin (14) argued that if the path dependence definition of David (7) allows a wide array of evolutionary processes, it must also allow a wide array of evolutionary outcomes. As a result, path dependence theory can be consistent with continuing forms of cumulative change and evolution. This developmental model allows forms of change over discontinuous change.

### Section (2): The Sociopolitical Alterations and the Health Sector in Egypt

Egypt’s public sector has been significantly affected by the sociopolitical alterations that took place during the second half of the last century. The health sector is no exception and has undergone several transformations and reforms that aimed at providing the Egyptian people with more equitable health coverage and accessible healthcare services.

#### The Sociopolitical Eras

The relationship or the repercussion of the socioeconomic alterations on the healthcare system will be analyzed in two major eras of Egypt’s modern history based on the classification provided by Saleh et al. (15). The two major eras are

1. The Post-Monarchy Socialist Era
2. The Privatization Era.

These two eras reveal the sociopolitical context; the reasons behind it; and the effects on the healthcare system during the reign of three Egyptian presidents and their governments up until the Arab Spring uprisings and beyond. Historically, the Arab world sustained several centuries under a non-self-rule of the Ottoman Empire followed by the European occupation until the emergence of strong independence movements. Egypt was no exception, as during the late nineteenth and early twentieth centuries, several uprisings took place, demanding the evacuation of the British occupation and its forces out of Egypt. Building on the idea of independence, the movements opposed the monarchy system in Egypt and eventually led to its topple in 1952 (15). While the healthcare system and financing mechanisms before 1952 are not documented, this period saw the establishment of the Ministry of Health in 1936, numerous public hospitals, and medical education institutions.

##### The Post-Monarchy Socialist Era

Following the overthrow of the monarchy in Egypt, the Arab Republic of Egypt was established by a group of military leaders, among them Gamal Abdel Nasser. The new post-monarchy political system revolved around the idea of Pan-Arab nationalism (also known as Nasserism) in attempts to achieve Arab unity. The state pushed the people and the institutions into this ideology through media, propaganda, legislations, and political instances. Attracted by the appeal of socialism, leaders of post-monarchy Egypt adopted many features of the Eastern Bloc social system, which led to strong economic, political, and military relations between Egypt and the Soviet Union (15). It is important to note that this choice of alliance with the East bloc serves as the critical juncture for the establishment of the current healthcare system in Egypt. This important alliance between Egypt and the Soviet Union had its implication on developing the healthcare system and the healthcare institutions in Egypt. The socialist approach of the political system in Egypt was interpreted into the notion of a welfare state, which was reflected in the legislation of free access of healthcare to all citizens in the Constitution. The notion of free access to healthcare came in line with the Egyptian people’s demands after toppling the monarchy and mandated a new health financing structures and policies (15). As a result, the new healthcare system was characterized by public ownership of all of the core system functions, such as fund collection, pooling, service purchasing, service provision, and regulation of services. All these functions were centralized under the state control, which in a way was the Government of Egypt’s (GoE) attempt to mimic the eastern Semashko model (16). Although the system adopted the notion of “free access to all citizens,” the system eventually resulted in financial unsustainability and fiscal deficits eventually, which in turn resulted in several weaknesses in the healthcare system such as shortages in supplies and medications (17, 18), poor wages for healthcare providers, poor maintenance of the public facilities, and moral hazards in over utilizing the services (19).

###### Number of Beneficiaries

It is important to note that during this post-monarchy era, the state embarked on developing the institutions and the infrastructure for the healthcare services, most notably the Health Insurance Organization (HIO) in 1964, under the notion of free access to health services to provide mandatory health insurance for the formal sector. However, of the 31 million Egyptians, the HIO pool by this time served only 14,000 beneficiaries, who were mainly government employees. This significant low ratio between the number of beneficiaries and population was at a standstill until the end of the Arab–Israeli war. This standstill status in insurance coverage was mainly due to the reallocation of state resources during the period of the Arab–Israeli war between 1967 and 1973 to fund military activities (20).

##### The Privatization Era

After the war against Israel and following peace treaty, the political approach under President Sadat changed and aimed at opening doors for private actors and investors to participate and contribute to the economy and promoting private and foreign investments, which is known as the “Infitah” policy (21). However, this new twist in the state policy was neither reflected in the healthcare system governance nor in the financing system, even during Mubarak’s tenure. By the late 1980s, the accumulated challenges of the financial unsustainability and the fiscal deficits in the health sector forced the state to look for alternative means to overhaul these problems (15). Accordingly, the GoE sought assistance and support from the international funding agencies, which by that time recommended to invest in the individual’s basic needs such as health, nutrition, and education based on privatization and role reduction of the public sector as per the Washington Consensus (22–24). According to these recommendations, there was an emphasis on improving macroeconomic stability and integration with the international economy through reforming fundamental elements, including public expenditure redirection, a tax scheme, interest rates, competitive exchange rates, financial and trade liberalization, liberalization toward foreign investment, privatization of state enterprises, and deregulation or a reduced role of the state (24). Accordingly, the World Bank released a report recommending the introduction of user fees or copayments as a possible solution for the fiscal deficits (25).

Egypt under Mubarak started progressively to implement the recommendations of the international funding agencies, which led to a gradual withdrawal of state subsidies for social programs and therefore, also led to a reduction in the public expenditure on health. The results backfired as it led to shortages in healthcare supplies, worsening of the quality, high out-of-pocket (OOP) payments, socioeconomic inequities, and passive privatization (15, 26–27). In addition, the recommendation of introducing user fees was found to be ineffective and constituted a barrier to accessibility, especially for the poor and vulnerable groups (28, 29). Accordingly, many countries and international agencies reconsidered the user fee and copayments recommendations and proposed the adoption of the pre-payment policy (to replace the user fee policy) and also recommended a gradual increase in the public expenditure on health (30, 31). However, Egypt did not comply with these recommendations and, in fact, did the opposite, as it remained true to its historical trend of low public expenditure on health and allocated fewer resources to the health sector despite the increased demand for health resulting from substantial population growth and a longer life expectancy. Consequently, the percentage of OOP payments rose significantly to reach 70% of the total health expenditure (32). In 2007, GoE endeavored to privatize the healthcare service through a decree for the establishment of the Egyptian Holding Company for Health with the intention to move all the governmental health insurance assets to the new company. However, the Egyptian Court of Administrative Justice negated the decree based on unconstitutionality (27, 33).

###### Number of Beneficiaries

Regarding the extension of the HIO coverage during this privatization phase (Sadat and Mubarak’s tenures), several legislations were enacted to achieve the notion of “free health for all.” During Sadat’s tenure, two laws stipulated the number of beneficiaries to the mandatory health insurance provided by HIO. As in 1975, Law 32 stipulated government employees to the mandatory health insurance and Law 79 that stipulated government, public and private sector employees, pensioners, and widows. In 1992, under Mubarak’s tenure, Law 99 stipulated extending insurance coverage to schoolchildren in a new program referred to as the School Health Insurance Program (SHIP). The SHIP was managed by HIO and the program managed to achieve universal coverage for schoolchildren by June 1995 (20). The exact number of beneficiaries under Sadat’s tenure is difficult to ascertain due to the scarce data in this period, but after law 99/1992 was passed, the numbers of beneficiaries under Mubarak’s tenure significantly increased to 36% of the total population by 1995 (20).

###### The Health Sector Reform Program (HSRP)

Later in 1997, GoE embarked on the HSRP. HSRP had a 9-year pilot phase, which was implemented in five governorates. HSRP targeted changes in the service delivery model and the financing mechanisms. The proposed financing model aimed to re-channel national health funds to local family health funds by imposing a mandatory health insurance with fixed premiums, copayments, and state subsidies for the poor (34). By the end of the pilot phase, official reports suggested some success in the shift from secondary to primary care in child treatment for fever/cough, as well as an increase in the child vaccination rate, an increase in the use of modern family planning, and a reduction in female malnutrition (35). However, other studies stated that there was significant population dissatisfaction with the services, financing model, and the financial burden compared with the services’ value (36).

###### The New Health Insurance Law

In 2009, another attempt to reform the health insurance system in Egypt was proposed by the government in the “New Health Insurance Law.” The most prominent component of this law was the introduction or increase in user fees, which in some services accounted for 25% of the service fees (*ILO Considerations on the Social Health Insurance Reform Project in Egypt*, 2009). The draft was debated in a parliamentary session in April 2010, but its implementation was postponed due to the Minister of Finance’s concerns at that time of the Egyptian government’s ability to finance the plan (15).

After its introduction, various drafts of the law have been subjected to various types of criticism, including the following:

- The 2009 official draft was mainly criticized for the unclear definitions of the benefits and the disproportionate increase in the financial burden for the population regardless of low incomes (37).
- The 2011 draft was criticized for the unclear contractual conditions, governance, quality indicators, and the unclear role of employers’ contributions to financing premiums for pensioners and widows (38).
- In 2013, the law draft was criticized for similar reasons as its previous versions and included unclear definitions, issues regarding contractual agreements and conditions, lack of detailed financing mechanisms, high formal user fees, increased contributions for children and schoolchildren, and an unclear mechanism of financing premiums for pensioners and widows (39).

From 2014 to the end of 2016, several drafts were proposed but never passed by the current House of Representatives, colloquially known as the Parliament. It is important to note that since the first draft of the law, 10 different governments have been in power, this high number due to the political events and turmoil over that period. Currently, the law continues to be debated and modified through national dialog. Nevertheless, it is unknown if and when the law will be passed despite repeated promises by the consecutive governments to finalize and pass it (40).

**Figure 1:**
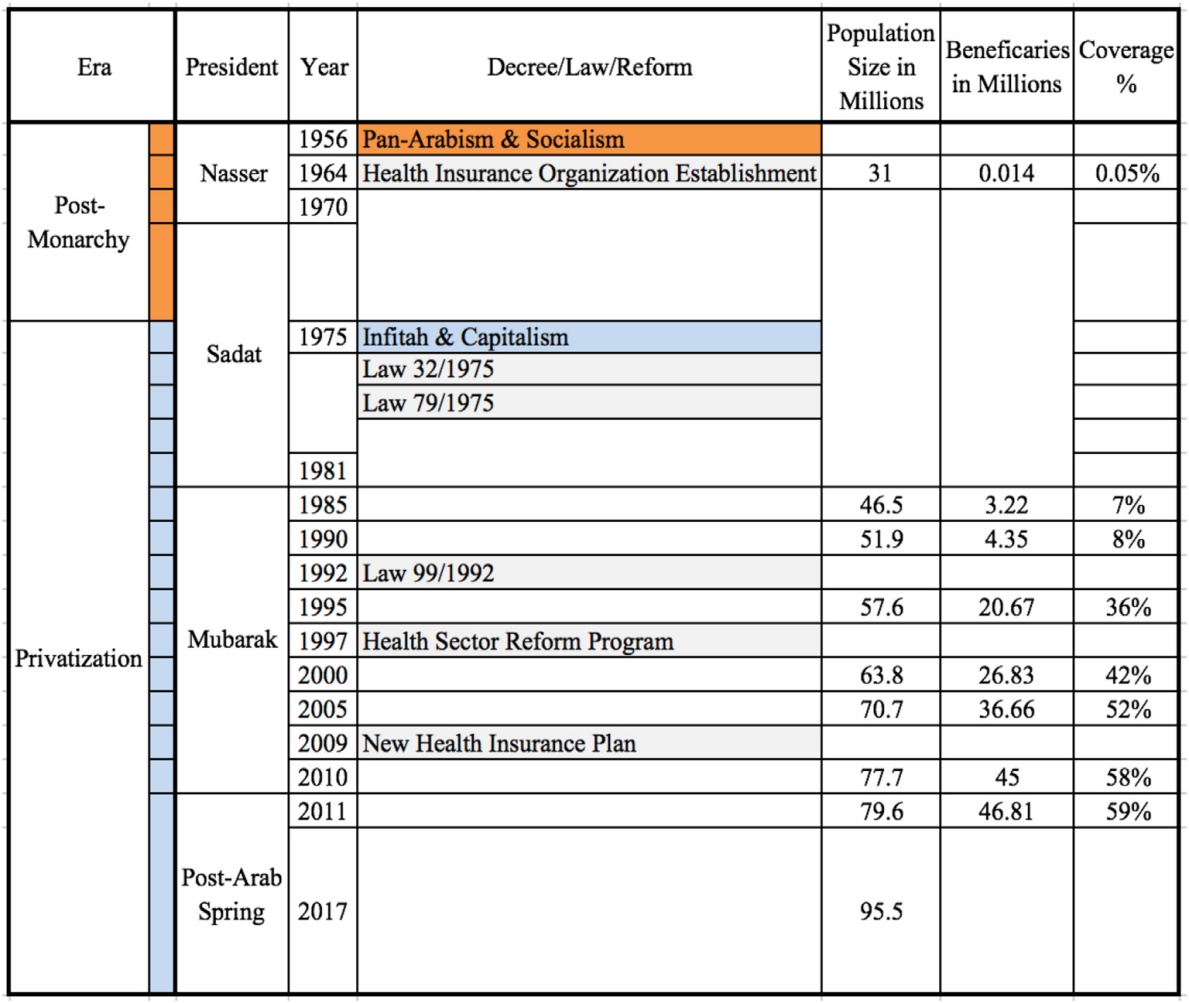
Reforms/decrees for extending health insurance in Egypt.

Figure 1 summarizes the major reforms/decrees in Egypt after the monarchy, by era, president, year, population census, beneficiaries of health insurance, and the coverage percentage. It is worth mentioning that information on the number of beneficiaries and the coverage percentage in 2017 is not available as shown in Figure 1; however, the significant increase in the population size in 2017 provides an idea about Egypt’s UHC challenge in the upcoming years.

## Discussion

As shown in the previous sections, combining literature on the development of UHC in Egypt and on path dependence theory can provide a possible explanation of the current status of the health sector based on the sociopolitical background of Egypt. Table 1 summarizes the analysis of the sociopolitical background and incorporates it into the structure of the health sector in Egypt based on Mahoney’s (8) analytical framework.

**Table 1:** The analytical structure of the Egyptian health sector’s path dependence.

Antecedent conditions	The conditions that led to the 1952 revolution and the sequential steps including the demands of the social rights, justice, moving away from the liberal monarchal system; the dispute with the western powers, and the rise of the pan-Arab nationalism

Critical juncture	Adopting the socialist approach and the establishment of the health institutions in a *centralized Soviet-influenced manner*, based on public ownership of the healthcare services functions (fund collection, pooling, service purchasing and service provision), “free health for all” notion, and tax-based financing mechanism

Structural persistence	The Soviet-influenced structure, the notion of free health for all, and tax-based financing based on formal employment has persisted up to the present, despite the change of the agency (presidents and cabinets) over the past 60 years

Reactive sequences	The plan to cover all Egyptians was largely limited to the formal sector and any attempts to extend the coverage targeted only the formal sector. Attempts to depart from the trajectory had limited or no positive effects. An inefficient taxation system coupled with low public spending on health led to insufficient funds for the health sector and the situation worsened with a significant increase in population

Outcome	As a result, the trajectory led to the current status of the health sector. It imposed several challenges for the healthcare financing system and the financial risk protection in Egypt, and thereby the overall health goals

Based on this review of the current status of the health sector in Egypt and its historical sociopolitical background, it is evident that the current unsatisfactory status of the health sector in Egypt is related to the path dependence of its structure. Outcomes of the path imposed numerous challenges in different aspects, mainly organizational and financial protection aspects.

### Organizational Aspect

#### Governance

The management of the health sector on both the macro- and the micro-levels suffers from a poor governance system due to the persistent Soviet Semashkho which is characterized by the centralized public ownership of the core financing system functions. Incompetent steering, ambiguous strategic planning, conflicted chain of authorities, and poor management of conflict of interests are not hard to notice for any observer. It is therefore essential to split between the financing system functions as in fund collection, pooling, service purchasing, and service providing in public institutions. This split will require reforming the institutional settings of the current health institutions in Egypt such as the Ministry of Health and Population and HIO or creating new independent institutions. Either way, new or existing institutions must take into consideration the concepts of governance, transparency, independency, and anti-corruption that can have positive outcomes in many aspects, most notably, the decision-making process, strategic planning, and the efficacy and efficiency of the services delivery (41).

#### Segmentation

Attempts to deviate from the contingent path have led to numerous complex segmentations regarding the funding bodies, which subsequently compromised the performance efficiency through high administrative costs, equity through segmenting the population into subgroups with different access, and quality of care through the lack of common quality standards due to the sub-sectors with different levels of quality of care (32).

### Financial Protection Aspect

#### The Poor and the Informal Sector

The reliance of the national tax-based health insurance on formal employment and the conventional top–down reform approach did not manage to extend the financial protection to the entire population and it neither managed to reach nor include the informal sector. The slight increase in the coverage percentage over the past few years can be attributed to the growth in the population in general, and newborns in particular.

#### Low Public Expenditure on Health

The sole reliance on the conventional top–down reforms accompanied with the economic difficulties and the weak taxation system led to the trend of low public spending in the health sector. Comparisons of the macroeconomic health indicators with other countries that possess similar population census, such as Turkey and Germany, show that Egypt is lagging behind (41). The low public expenditure reflects the state’s timid commitment toward the health sector.

#### High OOP Expenditure

As a result of the low public expenditure on health and the absence of adequate healthcare reforms, the percentage of OOP payments increased from 51% in 1995 to 71% in 2008 (42).

Path dependence theory has many definitions and interpretations. While different scholars have debated its usefulness in analyzing recent or past alternatives based on the antecedent conditions, they all agree that decision makers do not confront *tabula rasa* every time they have to make decisions and it is undeniable that past events exert some degree of limitations on current decisions. Therefore, path dependence theory can provide important insights through retrospective analysis of certain policies or events, which decision and policy makers can definitely leverage.

It is difficult for path dependence theory to explain all past events and alternatives. There is simply too much complexity in the structure and history of the organizations and the public policies across the world. In many countries, particularly the democratic ones, decisions are made based on dialogs and inputs from different stakeholders and might be reversed due to changes in cabinets, ruling parties, or other conditions. This complexity in democratic countries makes the evolutionary path more accepted than the continuous path. The conclusions drawn from using path dependence theory cannot be generalized to explain all public policies or organizational structures.

Based on the debates between the deterministic and the stochastic views and the debate between the canonical and the developmental models in “Section (1): Path Dependence Theory,” we believe that an interrelationship between the deterministic view and the canonical model exists and this interrelationship is manifested in terms of the continuity approach which both adopt. Another interrelationship also exists between the developmental model and the stochastic view in terms of adopting the evolutionary approach. The application of path dependence theory can vary from one country to another, depending on the governance, the available data, and the proper documentation of history.

The application of the theory should be idiosyncratic since it depends on the nature of each country and its political environment, the history of each organization, documentation, and the selected time span. We believe that path dependence theory is better used as a retrospective theory rather than a prospective one; it is better used to explain the past where all the variables have already taken place and naturally controlled rather than to expect the uncertain future where new inputs or variables can pose new reality and change the predicted trajectory. The theory is better used in its canonical model than the developmental model to limit the complexity. We also believe that the theory is more useful in countries or organizations where the decision-making process is centralized rather than decentralized. It can also be of greater use in democratic countries, where many stakeholders were involved in the process of decision-making since democracy can play a role in overturning previous policies and choices. For a country like Egypt, where the decision-making process and the hierarchy of its institutions were established in a centralized manner, path dependence theory can provide some answers and explanations to institutional structures and public policy.

## Conclusion

Path dependence theory provides a retrospective idea and explanation about the institutional structure and policies in many domains, including the health sector. It associates the sociopolitical antecedent conditions to the present status throughout several contingent chronologically ordered steps. The health sector in Egypt and its attempted reforms have followed a certain path based on its structure in the 1960s. This dependent path has resulted in the current unsatisfactory status of the system and particularly, it has yielded many challenges in the financial risk protection. Attempts to deviate from the contingent path that adopted a similar top–down reform approach have either failed or had minimal influence. The indicators of the financial risk protection in Egypt suggest that this path has reached its limit and that any attempts to continue on this path in the future will not yield any significant positive outcomes, especially in the light of the negligence of the growing and uninsured informal sector.

## Author Contributions

AF and FP equally contributed to the conception and development of the theoretical framework, writing of the manuscript, and editing of the subsequent drafts of the manuscript in light of the comments made by the reviewers for this journal.

## Conflict of Interest Statement

The authors declare that the research was conducted in the absence of any commercial or financial relationships that could be construed as a potential conflict of interest.
